# Dermal fibroblasts from long-lived Ames dwarf mice maintain their in vivo resistance to mitochondrial generated reactive oxygen species (ROS)

**DOI:** 10.18632/aging.100077

**Published:** 2009-07-31

**Authors:** Ching-Chyuan Hsieh, John Papaconstantinou

**Affiliations:** Department of Biochemistry and Molecular Biology, University of Texas Medical Branch, Galveston, Texas 77555, USA

**Keywords:** dermal fibroblasts, mitochondrial ROS, rotenone, 3-nitropropionic acid, antimycin A, thioredoxin, ASK1, p38 MAPK, resistance to ROS, Ames dwarf mouse, aging

## Abstract

Activation of p38 MAPK by ROS involves dissociation of an inactive, reduced thioredoxin-ASK1 complex [(SH)_2_Trx-ASK1]. Release of ASK1 activates its kinase activity thus stimulating the p38 MAPK pathway.  The level of p38 MAPK activity is, therefore, regulated by the balance of free vs. bound ASK1. Longevity of Ames dwarf mice is attributed to their resistance to oxidative stress.  The levels of (SH)_2_ Trx-ASK1 are more abundant in young and old dwarf mice compared to their age-matched controls suggesting that the levels of this complex may play a role in their resistance to oxidative stress.  In these studies we demonstrate that dermal fibroblasts from these long-lived mice exhibit (a) higher levels of (SH)_2_Trx-ASK1 that correlate with their resistance to ROS generated by inhibitors of electron transport chain complexes CI (rotenone), CII (3-nitropropionic acid), CIII, (antimycin A), and H_2_O_2_-mediated activation of p38 MAPK, and (b) maintain their in vivo resistance to ROS generated by 3NPA.  We propose that elevated levels of (SH)_2_Trx-ASK1 play a role in conferring resistance to mitochondrial generated oxidative stress and decreased endogenous ROS which are characteristics of longevity determination.

## Introduction

The Free Radical Theory of Aging proposes that endogenously produced oxygen radicals (ROS) are a basic cause of the progressive age-associated declines in tissue functions, and that oxidative stress generated by extrinsic environmental factors accelerate these declines [1-6].  Some of the biochemical characteristics of aged tissues are consequences of an increase in their pro-oxidant state, and it has been hypothesized that this affects the activity and function of key proteins of signal transduction pathways that regulate stress response [7-9].  The fact that ~90% of age-associated ROS originates from mitochondrial dysfunction emphasizes the importance of understanding the mechanism of mitochondrial ROS-linked stress signaling and its role in development of characteristics of aging and longevity [9,10].  Specifically, our studies and others have shown increased and stabilized basal levels of activities of the p38 MAPK and SAPK/JNK stress response signaling pathways suggesting that chronic ROS generated by mitochondrial ETC dysfunction may be a major causal factor that elevates the basal level of activity of these pathways in aged tissues [8-16].

**Figure 1.**
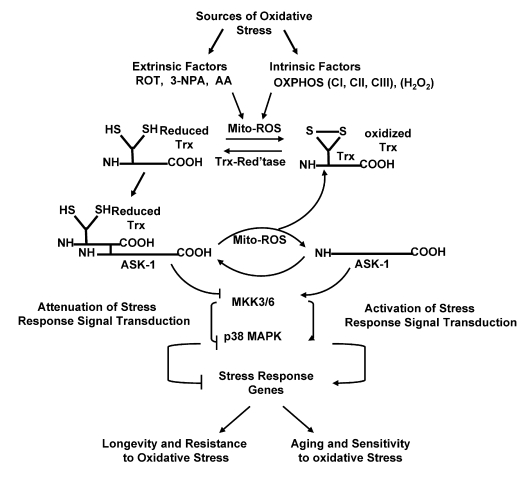
The Trx-ASK1 pathway of ROS mediated regulation of the p38 MAPK stress response pathway. The proposed hypothesis states that (**a**) the increased level of mitochondrial generated ROS in aged tissues/cells results in a locked-in cycle of ROS production and the amplification of oxidative stress signaling (p38 MAPK/SAPK-JNK). The chronic increase of endogenous levels of ROS stabilizes the increased basal, intrinsic changes in activity of regulators of the stress response which is the basis for the development of a state-of-chronic-stress and progressive decline in tissue function.  (**b**) Longevity and resistance to oxidative stress is associated with the elevated levels of the (SH)_2_Trx-ASK1 complex which attenuates the activity of stress response signaling  pathways (p38 MAPK/SAPK-JNK).  The levels of the (SH)_2_Trx-ASK1 complex decrease as the levels of endogenous ROS increase in aging tissues.  In long-lived mouse models the levels of the complex are elevated and part of the resistance to oxidative stress.

The mechanism that links ROS generated by dysfunctional mitochondria to the activation of the p38 MAPK and SAPK/JNK pathways involves regulation of the level of a reduced thioredoxin-ASK1 complex [(SH_2_)Trx-ASK1] (Figure 1).  This complex inhibits the activity of ASK1 and its activation of the downstream p38 MAPK and SAPK/JNK pathways [13,17].   In this mechanism the reduced form of thioredoxin [Trx (SH)_ 2_] interacts with the N-terminal domain of ASK1 *in vitro* and *in vivo,* thereby inhibiting the ability of this serine-threonine kinase of the MKKK family to activate the downstream components of both p38 MAPK and SAPK/JNK stress response signaling pathways [18,19].  Furthermore, formation of the (SH)_2_Trx-ASK1 complex occurs only with reduced Trx.  Thus, oxidation of the ASK1 bound Trx(SH)_2_ by certain oxidants including mitochondrial generated ROS mediates the dissociation of the complex and activation, by phosphorylation, of the free ASK1 [13].  Reduced Trx serves, therefore, as a physiological inhibitor of ASK1, and its oxidation and release from ASK1 links multiple cytotoxic stresses such as inhibitors of mitochondrial electron transport activity, H_2_O_2_, TNF, and Fas to activation of the p38 MAPK and SAPK/JNK stress response pathways [18-23].  We have proposed that the regulation of the ratio of ASK1: (SH)_ 2_Trx-ASK1 may be altered by ROS generated by mitochondrial electron transport chain (ETC) dysfunction.  Thus, the association and dissociation of the (SH)_ 2_Trx-ASK1 complex may be part of the molecular mechanism that elevates the endogenous activity of the p38 MAPK pathway in aged mice [11,14,16].  Furthermore, the persistent chronic increase in ROS production in aging tissue may be a basic factor that maintains and stabilizes the elevated level of signaling activity [11,12,14].  Using AML-12 hepatocytes in culture we have shown that the (SH)_ 2_Trx-ASK1 complex level is dramatically decreased in response to mitochondrial ROS generated by rotenone (ROT), an inhibitor of electron transport chain complex 1 (CI) [24,25], and that the level and activity of ASK1 and the basal level of activity of the kinases of the p38 MAPK stress response pathway are activated [13].

These studies suggest that ROS generated by the dysfunction of a specific component of the electron transport chain (ETC), *e.g.,* CI,  can activate the p38 MAPK stress signaling cascade via the dissociation of the (SH)_2_Trx-ASK1complex.  Studies with nematodes [26-29], *Drosophila* [30-33], and rodents [34-40] suggest that the molecular processes that regulate aging and longevity may be similar to those that regulate resistance to oxidative stress.  The longevity of the Snell and Ames dwarf mice and growth hormone receptor knock-out mice has been attributed to their resistance to oxidative stress [35,37,38].  This is supported by the observation that fibroblasts derived from these long-lived mice are significantly more resistant to ROS producing factors such as UV light, heavy metal (Cd), H_2_O_2_, paraquat and heat shock [37,41,42]. We have compared the *in vivo* levels of the (SH)_2_Trx-ASK1 complex in young vs. old controls to those in age-matched long-lived Snell dwarf mice and shown that the complex levels are significantly elevated in the dwarf livers and that the activities of the p38 MAPK pathway are significantly down regulated *in vivo* [13].  Similar results linking the ROS mediated regulation of p38 MAPK activity to the levels of the (SH)_2_Trx-ASK1 complex have been reported [19-21,23]. We have proposed that these characteristics, which are indicative of a decreased endogenous level of oxidative stress, may also be characteristics that confer resistance to oxidative stress to the long-lived mice.  In this mecha-nism the regulation of the (SH)_ 2_Trx-ASK1 levels is dependent upon the redox status of Trx (Figure 1).  Thus, the elevated levels of this complex are indicative of the decreased endogenous level of oxidative stress and may be a part of the mechanism of resistance to oxidative stress.  Our hypothesis is supported by the report that (a) activation of p38 MAPK in ASK1^(-/-)^ embryonic fibroblasts by H_2_O_2 _and TNF is abolished in these cells which exhibit resistance to these ROS producing stress factors [21]; and (b) the survival of Snell dwarf fibroblasts is associated with resistance to oxidative stress generated by UV light, heavy metal (Cd), H_2_O_2_, paraquat and heat [13,37,41,42].  These results raise the question of whether the levels of (SH)_ 2_Trx-ASK1 complex, which is redox sensitive, play a role in their resistance to oxidative stress and survival. Mechanistically, these studies suggest that the activity of uncomplexed ASK1 may be required for the sustained activities of p38 MAPK and SAPK/JNK [13,20,21,23].

In these studies we focus upon the role of ETC generated ROS on determination of the levels of (SH)_ 2_Trx-ASK1 complex and activation of the p38 MAPK pathway in fibroblasts from young (3-4 mos), middle aged (10-12 mos) and old (21-24 mos) wild type and Ames dwarf mice and whether this *in vivo* redox sensitive regulatory process is maintained in the fibroblast cell cultures.  Our studies address the potential role of the regulation of the (SH)_2_Trx-ASK1 complex levels in the mechanism of response of stress pathways to ROS generated by specific mitochondrial electron transport (ETC) dysfunction, which is a major physiological source of endogenous oxidative stress in aging tissues.  We propose that the mechanism by which long-lived mice exhibit characteristics of resistance to oxidative stress may involve the intracellular balance between free ASK1 vs. (SH)_2_Trx-ASK1 complex, that this mediates the level of activity of the stress response p38 MAPK and SAPK/JNK pathways, and is a basic difference between wild type and long lived mice.  To test our hypothesis, we correlate the levels of (SH)_2_Trx-ASK1 complex formation to the activity of the downstream p38 MAPK pathway, and resistance to oxidative stress in the Ames dwarf fibroblasts treated with rotenone (ROT), a specific inhibitor of ETC CI, 3-nitropropionic acid (3-NPA), a specific inhibitor of CII, antimycin A (AA), a specific inhibitor of CIII, and H_2_O_2_, a product of metabolism and inducer of oxidative stress, all of which mimic the generation of ROS by mitochondrial dysfunction [24,25,43].

## Results

### Growth curves of tail fibroblasts from young, middle aged and aged Ames dwarf mice 

**Figure 2.**
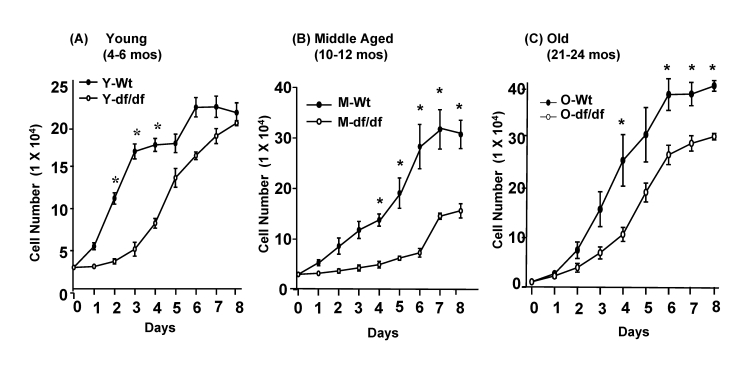
Growth curves of young (3-4 mos), middle aged and old (20-24 mos) wild-type and Ames dwarf fibroblasts. (**A**) Fibroblasts of young (3-4 mos) wild-type and Ames dwarf mice (3x10^4^); (**B**) middle aged (10-12 mos) wild-type and Ames dwarf mice (3x10^4^) and (**C**) old (20-24 mos) wild type and Ames dwarf mice (1x10^4^) were plated in triplicate in a 12-well culture plate. The cells from all cultures were harvested and counted daily for 8 days.  Each time-point represents the average number of cells in 3 triplicated wells of cultures established from 3 individual mice.  N = 3;  *p<0.05 between wild-type vs. dwarf mice.

Our previous studies have shown that the livers from young (3-6 mos) and old (20-23 mos) long-lived Snell dwarf male mice exhibit significantly higher endogenous levels of the Trx(SH)_2_-ASK1complex and lower p38 MAPK pathway activities than their age matched wild-type controls [13].  We interpreted these observations to indicate that the abundance of the complex is a determining factor for the maintenance of the lower endogenous levels of the p38 MAPK pathway activity and that this may be a physiological mechanism of the resistance to oxidative stress exhibited by these long-lived mice [13,34-39].  To facilitate further studies on the role of the Trx (SH)_2_-ASK1 complex as a part of the mechanism of the redox regulation of p38 MAPK activity and resistance to oxidative stress and in development of age associated characteristics we established fibroblast cultures from the tails of young (4-6 mos), middle aged (10-12 mos) and old (20-24 mos) wild-type and Ames mouse tail fibroblasts (Figure 2).   We plated the same number of fibroblasts in each well of a 12-well plate and counted the cells for 8 days to examine the growth patterns of wild-type and Ames dwarf fibroblasts.  The data show significant differences between the growth patterns of the wild type vs. young, middle aged and old dwarf fibroblasts.  For example, the wild type fibroblasts from all three ages, replicate at a faster rate than their age-matched Ames dwarf fibroblasts (Figure 2A-2C).  The fibroblasts from young and old dwarfs exhibit an initial lag period of ~3 days while the middle aged fibroblasts exhibit a lag period of ~6 days (Figure 2B).  The wild-type cells from all ages reached confluence at ~6 days.  However, the numbers of young wild type cells at confluence (~22 x 10^4^) are significantly lower than those of the confluent middle aged (30 x 10^4^) and old cell cultures (~40 x 10^4^). At the same time, a major difference in the growth of the Ames cells involves a lag period of ~ 3 days in both young and old cells after which time the young cells begin to replicate to attain a confluence of ~20 x 10^4^ at day 8. The middle aged cells, however, exhibit a 6 day lag period after which they replicate and reach a stationary phase (12 x 10^4^) which is considerably lower than the age-matched wild-type cells (Figure 1B).  The old dwarf fibroblasts reach confluency at day 7-8 (~30 x 10^4^) which is considerably lower than the corresponding wild type cells (~40 x 10^4^). In general, the growth characteristics of the Ames dwarf fibroblasts include an extended lag period, and a lower cell population at stationary phase.  There are no indications that any of these cultured cells, wild-type or dwarf, developed characteristics of transformation.

**Figure 3.**
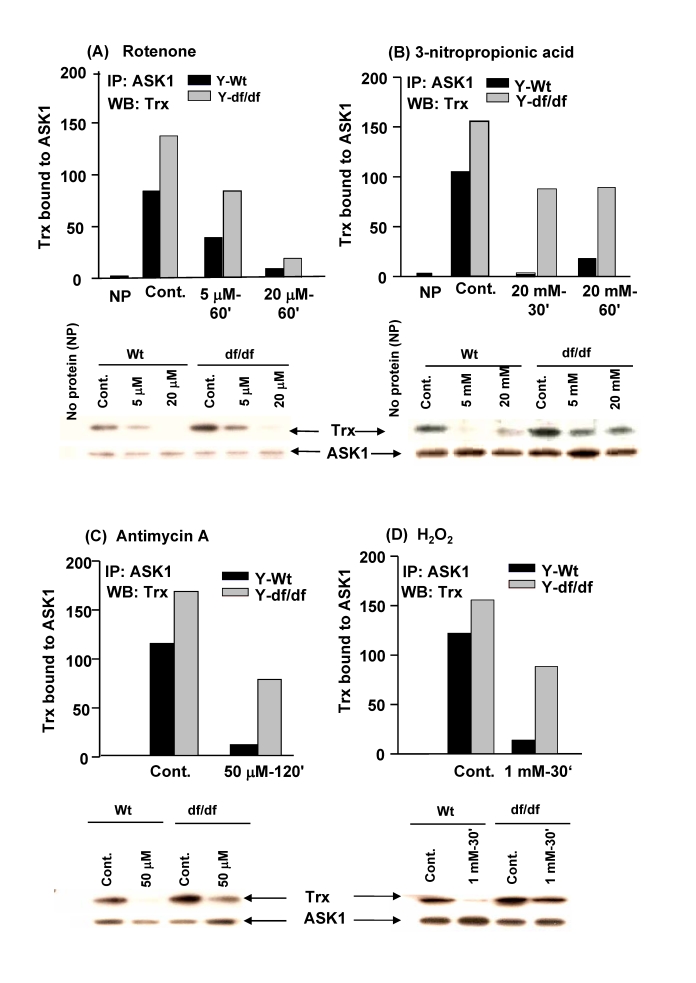
ROS generating inhibitors of electron transport chain complexes, CI, CII and CIII, and H _2_O_2 _affect the stability and levels of reduced thioredoxin-ASK1 complex [(SH)_2_Trx-ASK1]. The effects of: (**A**) CI inhibitor, rotenone, (5 μM and 20 μM for 60 minutes).  (**C**) CIII inhibitor, antimycin A, (50 μM for 120 minutes), and (**D**) H_2_O_2_ treatment (1 mM for 30 minutes) on the levels of the (SH)_2_Trx-ASK1 complex in young (4-6 mos) wild-type and dwarf fibroblasts.  Western blot analysis below each of the bar graphs (**A-D**) show the release of Trx bound to ASK1 as indicated by the amount of Trx co-immunoprecipitated with anti-ASK1 antibody after treatment with ROT, 3NPA, AA, and H_2_O_2_.

### Effects of ROS generating mitochondrial ETC inhibitors, ROT, 3-NPA, AA, and H_2_O_2_ on the stability and levels of the (SH)_2_Trx-ASK1 complex in young (4-6 mos) Ames dwarf fibroblasts

The mechanism of ROS-mediated activation of the p38 MAPK stress response pathway by ROT, an inhibitor of ETC CI that generates ROS in AML-12 hepatocytes, involves the release of ASK1 from the (SH)_2_Trx-ASK1 complex [13].  These results raised the question of whether ROS stimulated by specific mitochondrial ETC inhibitors may activate the p38 MAPK pathway via the same mechanism, *i.e.,* dissociation of the (SH)_2_Trx-ASK1 complex.  To address this we examined whether inhibitors of CI (ROT), CII ( 3-NPA), CIII (Antimycin A) and H_2_O_2_ stimulate the dissociation of this complex and p38 MAPK activity in wild-type vs. dwarf fibroblasts.  To examine the stability of the (SH)_2_Trx-complex in response to these factors we measured the levels of the intact complex by co-immunoprecipitation.  The data in Figure 3A show that the amount of Trx co-immunoprecipitated with anti-ASK1 antibody in wild-type cells  is severely decreased by treatment with 5 μM and 20 μM ROT for 60 minutes (Figure 3A).  A similar treatment of the dwarf cells also caused a decrease in the complex level but not as severe as in the wild-type cells.  The data also show an attenuated response to 20 mM 3-NPA, an inhibitor of CII (Figure 3B), 50 μM antimycin A (AA), an inhibitor of CIII (Figure 3C), and 1mM H_2_O_2_ (Figure 3D).  These data show that the basal level of the (SH)_2_Trx-ASK1 complex is significantly higher in the young dwarf fibroblasts vs. their age-matched wild type controls, and that dissociation of the complex is not as extensive in the dwarf cells as in the wild-type cells in response to these ETC inhibitors.

### Pool levels of reduced thioredoxin are higher in Ames dwarf fibroblasts

**Figure 4.**
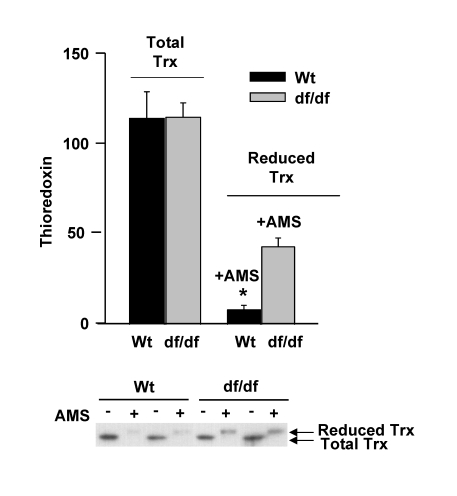
The pool levels of total thioredoxin and re-duced thioredoxin in fibroblasts derived from young wild type and dwarf mice. Cytoplasmic extracts (100 μg) of young wild type and dwarf fibroblasts were either untreated or treated with 4-acetamido-4'-maleimideyl-stilbene-2,2'-disulfonic acid (AMS) for 2 hours, resolved by15% SDS-PAGE, and transferred to PVDF membrane.  The Western blots shown are of two individual samples that detect the pool levels of total and reduced Trx. Western blot analyses show the level of reduced Trx and total Trx in young wild-type and dwarf fibroblasts.  The upper band represents reduced Trx pool levels; the lower band represents total Trx pool levels.  A similar pattern has been observed in three separate experiments.  * p<0.05 between wild type and dwarf.

The data in Figure 3 suggest that the levels of reduced thioredoxin may be higher in the dwarf fibroblasts.  This could explain the higher levels of the (SH)_2_Trx-ASK1 complex in the dwarf cells. To demonstrate that the dwarf fibroblasts are in a more reduced state than those from wild type mice, we treated the extracts of young wild type and dwarf fibroblasts with a thiol-reactive probe, 4-acetamido-4'-maleimidyl-stilbene-2,2'-disulfonic acid (AMS), to measure the level of Trx in these fibroblasts.  The binding of the thiol probe to reduced Trx causes a decreased mobility of the modified Trx in PAGE gels.  The results show the dwarf fibroblasts have an approximately 5-fold higher level of the reduced form of Trx as in the wild-type fibroblasts (Figure 4),  and that the relative proportion of the reduced Trx vs. total pool level of Trx is about 38% in dwarf and 7.5% in wild type fibroblasts.  This may explain the decreased activation of ASK1 and p38 MAPK in the dwarf fibroblasts.  These data are consistent with the results in Figure 3 that show the higher levels of (SH)_2_Trx-ASK1 complex in the dwarf cells and their resistance to oxidative stress.

### The effects of ROT on thioredoxin pool levels

**Figure 5.**
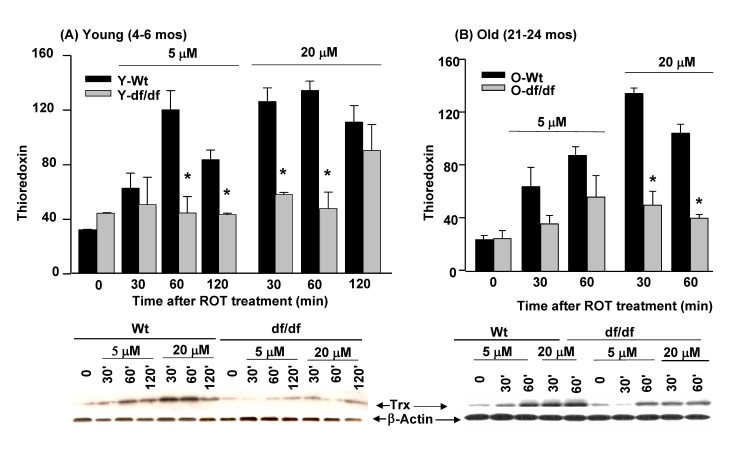
The effects of ROT (5. μ**M and 20 **μ**M) on the induction of thioredoxin in fibroblasts derived from young (4-6 mos) and old (21-24 mos) wild-type and Ames dwarf mice.** (**A**) Fibroblasts from young wild type and age matched dwarf mice.  (**B**) Fibroblasts from old wild type and age matched dwarf mice.

Analysis of the effects of ROT on total Trx pool levels in young wild type and dwarf fibroblasts show that there is an increase in pool levels of the wild type  (~4- fold) cells after both 5 μM and 20 μM treatment (Figure 5).  The Trx levels peak at 60 minutes in response to 5 μM ROT and appear to have initiated recovery at 120 minutes.  The increase in response to 20 μM ROT peaks more rapidly, *i.e.,* at 30 minutes after treatment, but does not appear to have entered a recovery phase at 120 minutes, suggesting a prolonged response to the inhibitor.  These data suggest that the concentration of the challenging agent determines the rapidity and extent of induction of Trx.  Interestingly, the intensity of the response is the same for both 5 μM and 20 μM ROT.  On the other hand, the dwarf fibroblasts do not respond to 5 μM ROT and exhibit a delayed induction of Trx at 120 minutes of treatment with 20 μM ROT.  The failure of the dwarf cells to respond to 5 μM ROT suggests that these cells are resistant to oxidative stress.

Similarly, treatment of old wild type and dwarf cells with 5 μM and 20 μM ROT also induced an increase in Trx pool levels.  These experiments showed that the pool levels of old wild type dwarf fibroblasts are induced to a greater extent by 5 μM and 20 μM ROT than the age-matched dwarf cells.  The induction peaks at 60 minutes in response to 5 μM ROT and at 30 minutes in response to 20 μM ROT.  The levels of Trx induction in old dwarf fibroblasts are lower at both ROT concentrations, although there is an increase at 60 minutes in response to 5 μM ROT.   We interpret these results to indicate that the decreased level of response of Trx in dwarf cells may be an indication of lower levels of oxidative stress by ROT treatment.

### The ROT mediated activation of p38 MAPK is attenuated in fibroblasts from young (4-6 mos) and old (21-24 mos) Ames dwarf mice 

The data in Figure 3 indicate that the dissociation of the (SH)_2_Trx-ASK1 complex is more resistant to ROT in the young dwarf fibroblasts suggesting that this may be a factor in the attenuated activation of the p38 MAPK pathway in the young and aged dwarf cells.  Phosphorylation of the amino acid residues of the p38 MAPK catalytic site, Thr^180^Tyr^182^, is indicative of the level of activity of this stress response signaling protein.

**Figure 6.**
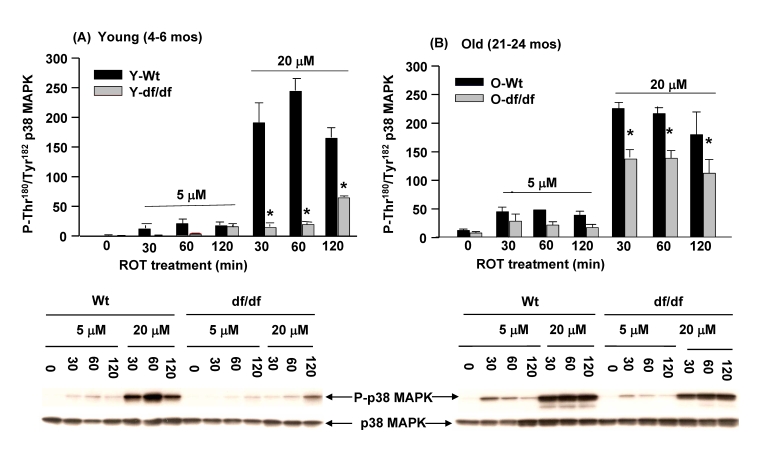
Fibroblasts from young (4-6 mos) and old (21-24 mos) dwarf mice show resistance to rotenone-induced p38 MAPK activation. Induction of phosphorylation of the p38 MAPK catalytic site amino acid residues (P-Thr^180^/P-Tyr^182^) by ROT is significantly lower in fibroblasts derived from young and old dwarf mice.  (**A**) The time course of activation of the p38 MAPK catalytic site by rotenone (5 μM and 20 μM) in fibroblasts from young (4-6 mos) wild-type and Ames dwarf mice shows the differences in levels of response to 5 μM and 20 μM ROT.  (**B**) A time course of the activation of old (21-24 mos) wild-type and dwarf mouse fibroblasts in response to 5 μM and 20 μM ROT shows that the level of activation of p38 MAPK catalytic site in aged wild-type and dwarf fibroblasts increases with age.  The Western blot time course analyses below each bar graph show that the phosphorylated levels of the p38 MAPK catalytic site are significantly lower in Ames dwarf fibroblasts than their sage matched wild-type controls.  n = 4; * p<0.05 between wild-type and dwarf.

In previous studies we showed that the basal levels of p38 MAPK phosphorylation and its kinase activity are significantly lower in the age-matched Snell dwarf mice [13].  These results suggested that the level of activity of the p38 MAPK pathway may be subject to the redox state of the cells and may be indicative of decreased endogenous oxidative stress associated with the longevity of this mouse [9,13,35,37]. To address this we measured the levels of ROT mediated phosphorylation of the p38 MAPK catalytic sites, Thr^180^Tyr^182^(Figure 6). The data show that in young and old Ames fibroblasts and their age-matched wild-type controls the response to 5 μM and 20 μM ROT is significantly greater in the young and old wild type cells than in the age-matched young and aged dwarf cells (Figure 6A, 6B).  Notably, the responses to 5 μM and 20 μM ROT by young and old wild type fibroblasts show a similar level of activation of p38 MAPK.   However, a major difference that occurs with the old dwarf cells involves their response to  20 μM ROT at 30 minutes which suggests these cells from old Ames dwarf mice have become more sensitive to oxidative stress as they aged.  Although these results suggest that the lower level of inducibility of the p38 MAPK catalytic site phosphorylation in young and aged dwarf fibroblasts may be a characteristic of resistance to oxidative stress, the old dwarf cells also show a significant increase in sensitivity to the 20 μM ROT.

### The antioxidant N-acetyl cysteine (NAC) attenuates the ROT mediated induction of p38 MAPK catalytic site phosphorylation

**Figure 7.**
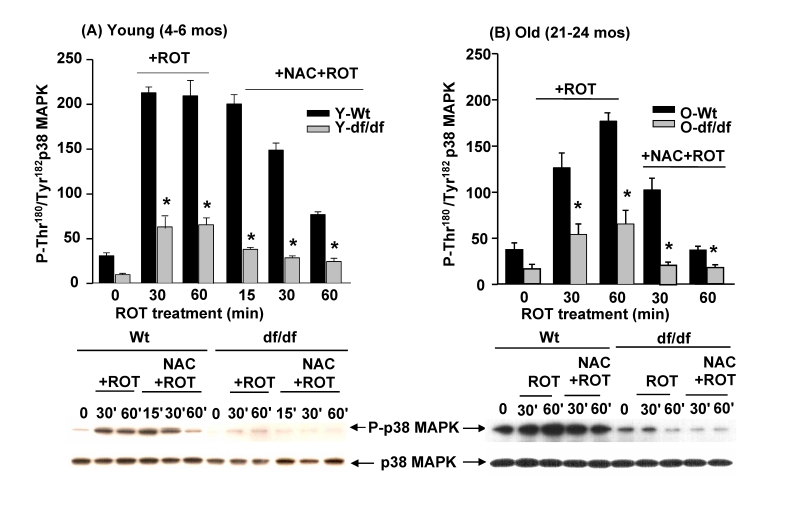
The antioxidant, N-acetyl cysteine (NAC) attenuates the ROS-mediated activation of p38 MAPK by ROT in young and old wild-type and Ames dwarf fibroblasts. The bar graphs and Western blot analyses below each bar graph show a time course of the effects of ROT (20 μM and NAC + ROT treatment on the induction of phosphorylation of the p38 MAPK catalytic site residues, P-Thr^180^/P-Tyr^182^, in young (4-6 mos) wild-type and Ames dwarf fibroblasts, and (B) in old (21-24 mos) wild type and Ames dwarf fibroblasts.  n=4;  * p<0.05 between wild-type and dwarf.

Our previous studies have shown that ROT treatment of AML 12 hepatocytes in culture stimulates the dissociation of the (SH)_2_Trx-ASK1 complex and that this results in activation of p38 MAPK [13].  These studies have also shown that treatment with the antioxidant N-acetyl cysteine (NAC) prevented the dissociation of the complex and activation of p38 MAPK.  A similar experiment shows that the ROT activation of p38 MAPK in young dwarf fibroblasts is further decreased by NAC treatment (Figure 7A.)  These data show a ~7-fold induction of p38 MAPK catalytic site phosphorylation by 30 minutes of ROT treatment that remains at the peak level for 60 minutes (Figure 7A).  Furthermore the data show a significantly lower response to ROT by NAC treated wild-type cells (Figure 7A).  A similar treatment of young dwarf fibroblasts shows a lower response to ROT and an attenuation of that response by NAC cells.  These results suggest that the ROT mediated activation of the p38 MAPK is a redox-sensitive reaction that is attenuated by the antioxidant treatment of fibroblasts from young and old wild-type and dwarf mice.

### ROT-mediated activation of ATF-2 activity is attenuated in Ames dwarf fibroblasts

**Figure 8.**
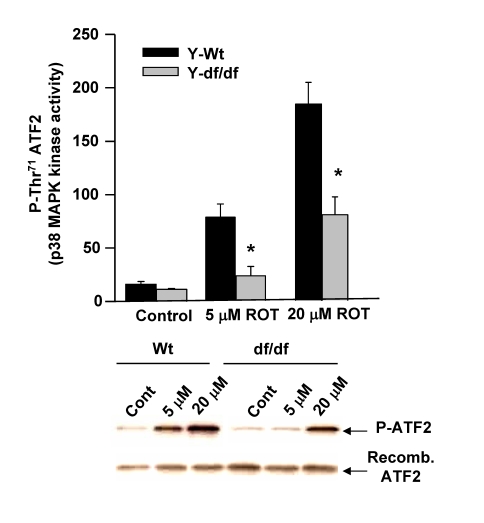
The Ames dwarf fibroblasts show a decreased level of induction of p38 MAPK kinase activity in response to ROT. Extracts from young wild type and dwarf fibroblasts were used to measure the p38 MAPK kinase activity using the Thr71 residue of ATF-2 as its substrate.  The bar graphs and immunoblots show the effects of 5 μM and 20 μM ROT on the *in vitro* p38 MAPK kinase activity as indicated by the level of induction of phosphorylation of the Thr71 ATF-2 catalytic site amino acid residue.

Our past studies have indicated that the elevated basal level of p38 MAPK kinase activity in nuclei of old mouse livers mediates an increased activation of ATF-2 via the phosphorylation of its catalytic site at Thr^71^ and that this activity is significantly lower in both young and aged Snell dwarf liver nuclei [13]. Since ATF-2 is a transcription factor activated by p38 MAPK we used this reaction as an indicator of stress associated activity of P-p38 MAPK in ROT treated young wild-type and dwarf fibroblasts.  To demonstrate the differences in response to ROT by the wild-type vs. dwarf fibroblasts we measured the *in vitro* phosphorylation of Thr^71^ of  recombinant ATF-2 in wild-type and dwarf cell extracts.  The data in Figure 8 show that phosphorylation of ATF-2 Thr^71^ is induced by treatment of the wild-type and dwarf fibroblasts with 5 μM and 20 μM ROT for 60 min.  Although the endogenous nuclear levels of p38 MAPK phosphorylation of the ATF-2 Thr^71^ are similar in the wild-type and dwarf fibroblasts, treatment of the wild type fibroblasts with 5 μM ROT resulted in ~4-5-fold induction of the Thr^71^ phosphorylation and a ~10-fold induction by 20 μM ROT.  These results showed that the kinase activity in ROT treated dwarf fibroblasts is significantly lower than their age-matched wild-type controls.  The attenuated activity of nuclear P-p38 MAPK correlates with the decrease in ATF-2 activation and with the elevated levels of (SH)_2_Trx-ASK1complex in the dwarf fibroblasts.  Our data suggest that the attenuated targeting and activation of ATF-2 in the dwarf fibroblast is indicative of their resistance to oxidative stress.

### Activation of p38 MAPK by 3-NPA, an inhibitor of complex II (succinic dehydrogenase) is attenuated in Ames dwarf fibroblasts

**Figure 9.**
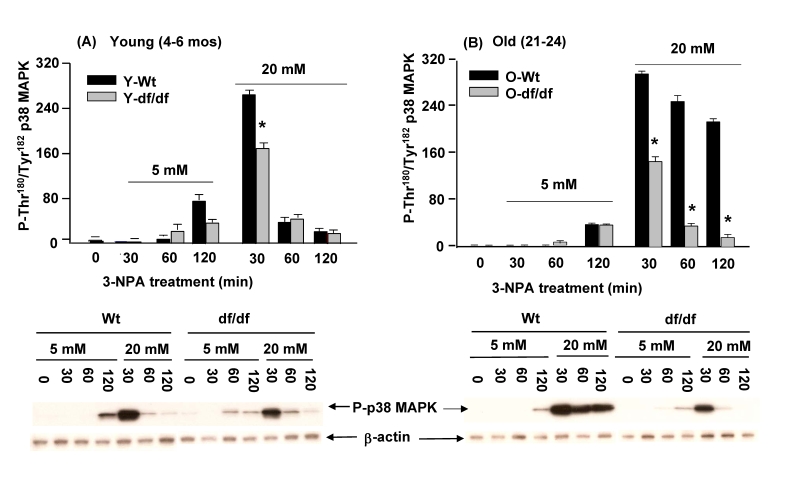
Effects of 3-NPA, an inhibitor of CII (succinic dehydrogenase) activity on the phosphorylation of the p38 MAPK catalytic site in fibroblasts from young (4-6 mos) and old (21-24 mos) wild-type and dwarf mice. (**A**) The bar graphs and western blot analyses below each bar graph show the phosphorylation levels of the p38 MAPK catalytic site amino acid residues (P-Thr^180^/P-Tyr^182^) in (**A**) young fibroblasts (4-6 mos) and (**B**) old wild-type and dwarf fibroblasts after treatment with 5 mM or 20 mM 3-NPA.  The fibroblasts from young dwarf mice show an attenuated response to the 3-NPA-mediated phosphorylation of the catalytic site (n=3).  Both young and old wild-type and dwarf fibroblast responses peak at 30 minutes.  However, the recovery of the old wild-type cells is delayed.  The fibroblasts from old dwarf mice also show resistance and rapid recovery to 3-NPA treatment similar to the response shown by the young dwarf fibroblasts.  *represents p<0.05 between wild-type vs. dwarf.

In recent studies we demonstrated that 3-NPA, an inhibitor of succinic dehydrogenase at CII of the mitochondrial ETC generates ROS at a site between the ubiquinol pool and the 3-NPA block in CII [43].  Furthermore, we demonstrated that the basal kinase activities of redox-sensitive p38 MAPK, JNKs, MKK4, MKK7, MKK3 and ATF-2 are all elevated in hepatocytes of 3-NPA treated C57Bl/6 mice [10,14].  Since 3-NPA stimulates ROS production and results in p38 MAPK and SAPK/JNK pathway activation we conducted experiments to determine whether the wild-type vs. dwarf fibroblasts, young vs. old, exhibit altered levels and duration of response to 3-NPA.  Our goal is to determine whether the resistance to oxidative stress in the Ames fibroblasts includes the ROS generated in response to this CII inhibitor.  The differences in response to 3-NPA *i.e.,* phosphorylation of p38 MAPK catalytic site residues by the fibroblasts from young and old wild type and dwarf mice are shown in Figure 9.  These data show that phosphorylation of the p38 MAPK catalytic site in young and old wild-type cells is detected at 120 minutes in response to 5 mM 3-NPA, while treatment with 20 mM 3-NPA peaked at 30 minutes (Figure 9A and 9B).  As with ROT, the intensity of the response is significantly lower in both young and old dwarf fibroblasts.  In fact, a comparison of the responses by young and old dwarf fibroblasts to both 5 mM and 20 mM 3-NPA shows that the intensities of their peak responses are similar (Figure 9A and 9B).  On the other hand, a significant difference is indicated by a prolonged response by old wild-type fibroblasts to 20 mM 3-NPA.  Thus, although the intensity of the response was essentially the same in cells of both ages, the recovery of the young wild-type cells was rapid, *i.e.,* at 60 minutes of treatment whereas the response by the old wild-type fibroblasts which also peaked at 30 min. was sustained for up to 120 minutes, twice the time of the recovery of the young fibroblasts.  We interpret this to indicate that the Ames fibroblasts, young and old exhibit a similar level of resistance to CII (succinic dehydrogenase) dysfunction.

### Activation of p38 MAPK by antimycin A (AA), an inhibitor of complex III is attenuated in Ames dwarf fibroblasts

**Figure 10.**
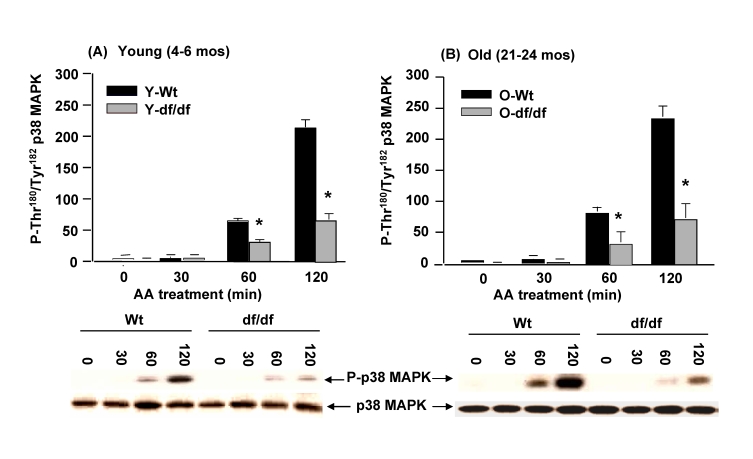
The young and old Ames dwarf fibroblasts show an attenuated phosphorylation of p38 MAPK catalytic site in response to antimycin A. The bar graphs and western blot analyses below each bar graph show the pool levels and the levels of phosphory-lation of the p38 MAPK catalytic site amino acid residues (P-Thr^180^/P-Tyr^182^) in (**A**) young wild-type and dwarf fibroblasts (4-6 mos) and (**B**) old wild-type and dwarf fibroblasts  (21-24 mos) after treatment with 50 μM AA.   The fibroblasts from young dwarf mice show an attenuated response to the AA-mediated phosphorylation of the catalytic site.  The fibroblasts from young and old dwarf mice show similar levels of resistance to AA suggesting that resistance to CIII generated ROS does not change with age in these fibroblasts.  n=3, *p<0.05 between wild-type vs. dwarf.

CIII, which has been identified as a key site for ROS generation [44], has two centers: the Q_0_ center, oriented toward the intermembrane space and the Q_i_ center, located in the inner membrane and facing the mitochondrial membrane.  Antimycin A (AA) inhibits CIII at the Q_i_ center and increases superoxide generation at the Q_0_ center.  In order to analyze the effects of ROS generated by CIII dysfunction we treated the fibroblasts with 50 μM AA for 120 minutes.  The data in Figure 10A and 10B show that p38 MAPK catalytic site phosphorylation is activated in both young and old wild-type fibroblasts and that the kinetics of activation are similar at both ages.  The data also show that AA mediated induction of phosphorylation of the p38 MAPK catalytic site follows the same pattern of activity, but the level of induction is significantly lower in young and old dwarf fibroblasts compared to the wild-type cells.  These data suggest that the level of sensitivity of the wild type and dwarf fibroblasts remains the same in both young and old, and that this response differs from the response to ROT which becomes less resistant in old dwarf cells.

**Figure 11.**
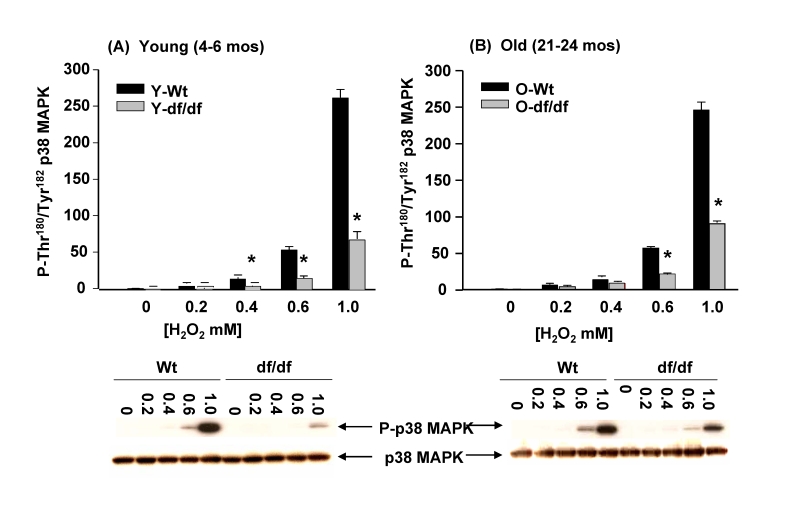
The effects of H _2_O_2_ on the phosphorylation of p38 MAPK catalytic site in wild-type and Ames dwarf mouse fibroblasts. The fibroblasts from young (4-6mos) and old (21-24 mos) wild type and dwarf mice show an attenuated induction of phosphorylation of the p38 MAPK catalytic site amino acid residues in response to H_2_O_2_.  The bar graphs and western blot analyses below each bar graph show the pool levels and levels of phosphorylation of the p38 MAPK catalytic site amino acid residues (P-Thr^180^/P-Tyr^182^) in (**A**) young wild-type and dwarf fibroblasts (4-6 mos) and (**B**) in old wild-type and dwarf fibroblasts (21-24 mos) after treatment with varying concentrations of H_2_O_2_ (0.2 to 1.0 mM). The p38 MAPK activities of both young and old wild type fibroblasts continue to rise exponentially up to the highest level of H_2_O_2_ (1 mM) treatment. The fibroblasts from young and old dwarf mice show similar levels of resistance to increasing levels of H_2_O_2_ suggesting that resistance to this ROS species does not change with age in these fibroblasts.  n=3, * p<0.05 between wild-type vs. dwarf.

### Activation of p38 MAPK by H_2_O_2_ is attenuated in Ames dwarf fibroblasts

H_2_O_2_ has been used as a measure of CI and CIII as well as extra-mitochondrial sites of ROS production.  The data in Figure 3D show that 1mM H_2_O_2_ treatment for 30 minutes mediates almost complete dissociation of the (SH)_2_Trx-ASK1 complex in the young wild-type fibroblasts.  On the other hand the dwarf cells show a significant resistance to the same treatment.  Thus, although the basal levels of the complex are similar in wild-type and dwarf cells, its dissociation is significant-ly attenuated. These results suggest that the H_2_O_2_-mediated induction of phosphorylation of the p38 MAPK catalytic site may involve dissociation of the complex and that this response to H_2_O_2_ is attenuated in the dwarf.  To address this question we measured the levels of activation of p38 MAPK by varying concentrations of H_2_O_2_.  The data in Figure 11A and 11B show that the inducibility of p38 MAPK catalytic site phosphorylation is detectable by 0.4 mM H_2_O_2_  and increases dramatically from 0.6 mM to 1 mM H_2_O_2_. On the other hand, similar treatment of the young and old dwarf cells show that the response to all H_2_O_2_ concentrations is significantly attenuated.  The responses of wild-type and dwarf fibroblasts to H_2_O_2_ correlate with the levels of dissociation of the (SH)_2_Trx-ASK1 complex.   Our data suggest that the dwarf fibroblasts are resistant to H_2_O_2_mediated oxidative stress.

### Differences in sensitivity of young, middle aged and old Ames fibroblasts to increasing concentrations of ROT: The age-associated progressive loss of resistance to ROT mediated oxidative stress by dwarf fibroblasts

**Figure 12.**
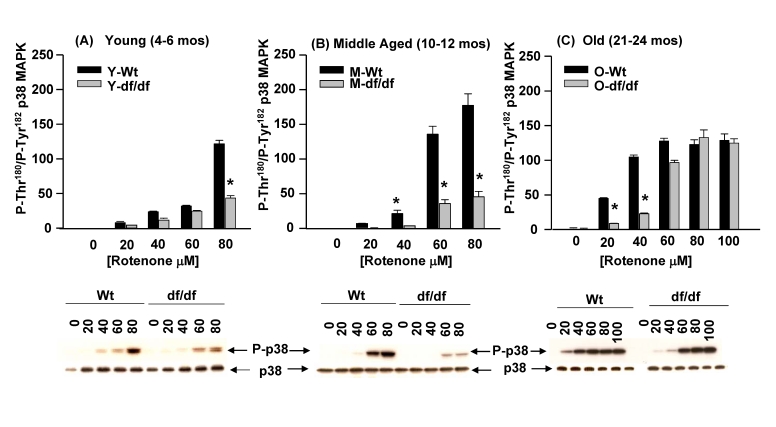
The effects of increasing concentrations of rotenone on the activation of p38 MAPK phosphorylation in young (4-6 mos), middle aged (10-12 mos) and old (21-24 mos). The bar graphs and western blot analyses below each bar graph show the pool levels of P-Thr^180^/P-Tyr^182^ p38 MAPK in (**A**) young (4-6 mos);  (**B**) middle aged (10-12 mos) and  (**C**) old (21-24 mos) wild-type and dwarf fibroblasts after treatment with increasing concentrations of ROT for 30 minutes.  n=3, *p<0.05 between wild-type vs. dwarf.

The data in Figure 6B (ROT) indicate that the old dwarf cells develop a decreased level of resistance in response to both 5 mM and 20 mM ROT. The data in Figure 12 show an extension of these experiments in which we measured the effects of increasing concentrations of  ROTfrom 20 mM to 100 mM on the phosphorylation of the p3 p38 MAPK catalytic site residues in young, middle aged and  old wild-type and dwarf  fibroblasts. The data clear-ly show an increased sensitivity of the old wild-type cells in response to 20 mM ROT and 40 mM ROT not seen in the young and middle aged cells.  The aged dwarf fibroblast response peaks at 60 mM ROT;  the middle aged fibroblasts show increased sensitivity to 60 μM ROT and peaks at 80 μM ROT and the young wild type cells show resistance up to 60 μM and a strong response to 80 μM ROT.  These data suggest that the wild-type cells develop an age-associated progressive increase in sensitivity to ROT.  The dwarf fibroblasts also show a progressive increase in sensitivity to ROT which is evident at 60 μM in the old fibroblasts.  The responses of the young and middle aged dwarf fibroblasts are essentially the same suggesting that they retained their resistance to oxidative stress up to that age.  However, a significant increase in sensitivity occurs in the old dwarf fibroblasts in response to 60 μM ROT.  It is at the 80 μM and 100 μM ROT levels that the responses of the wild type and dwarf fibroblasts are the same.  These data support our hypothesis that both wild-type and dwarf fibroblasts develop age-associated sensitivity to ROT, but ROT mediated the development of this sensitivity is delayed in the dwarf fibroblasts.  These experiments suggest that resistance to oxidative stress is lost with age, by wild-type and dwarf fibroblasts, but this loss is delayed in the dwarf cells.

## Discussion

Approximately 90% of age-associated ROS production is of mitochondrial origin due to ETC dysfunction [10].  This emphasizes the importance of understanding the molecular mechanism of ROS-linked activation of stress signaling pathways and the role of this physiological status in the development of characteristics of aging and longevity such as sensitivity and resistance to oxidative stress [9].  Our proposed mechanism for the activation of the p38 MAPK (and SAPK/JNK) stress response pathways by ROS generated by ETC dysfunction is supported by the demonstration that ROS generated by ROT induced CI dysfunction activates this stress response pathway [13].

These studies suggested that the state of oxidative stress caused by dysfunction of CI, CII and/or CIII as well as H_2_O_2_may activate the p38 MAPK stress response pathway, and may be a chronic physiological characteristic that plays an important role in the development of physiological characteristics of aging [9].  In the present studies we demonstrated that inhibitors of specific ETC activities, *i.e.,* CI, CII and CIII, and H_2_O_2_ activate the p38 MAPK stress response pathway. We propose that the mechanism of this activation involves the ROS mediated release of ASK1 from an (SH)_2_Trx-ASK1 complex thereby enabling ASK1 to activate the p38 MAPK and SAPK/JNK pathways (Figure 1) [13, 18-21, 23].

Our past studies have demonstrated that the levels of the (SH)_2_Trx-ASK1 inhibitory complex are significantly higher in the livers of young and old Snell dwarf mice than in their age-matched controls.  Since these long-lived mice exhibit an endogenous resistance to oxidative stress we proposed that the level of (SH)_2_Trx-ASK1 may be a physiological characteristic of  longevity.  This hypothesis is strongly supported by the studies presented here, which have shown that in the dwarf fibroblasts (a) the levels of the (SH)_2_Trx-ASK1 complex and reduced Trx are significantly higher; (b) the dissociation of the complex by ROT is attenuated in both young and old Ames dwarf cells.  These mice also exhibit an extended life span and resistance to oxidative stress.  Our proposal that the level of the (SH)_2_Trx-ASK1 complex is indicative of and part of the mechanism of resistance to oxidative stress is supported by the fact that the dissociation of the complex is much more complete in response to the specific inhibitors of CI, CII, and CIII activity and to H_2_O_2_  in the wild-type cells than in age-matched dwarf cells.

Using cultured Ames dwarf fibroblasts has revealed that the *in vivo* mechanism of activation of the p38 MAPK pathway is not altered by their *in vitro* environment (tissue culture).  Thus, the regulation of the level of (SH)_2_Trx-ASK1 complex formation and dissociation, and resistance to the oxidative stress generated by the ETC complexes is retained in the cultured dwarf fibroblasts.  Furthermore, this *in vivo* characteristic is maintained in the fibroblast for 50 passages as indicated by their continued resistance to H_2_O_2_.  We propose, therefore that the mechanism of resistance to oxidative stress in the dwarf fibroblasts may be an epigenetic physiological process which is established *in vivo* and is maintained *in vitro.*

Our proposed mechanism is supported by the response of the young ROT treated dwarf fibroblasts to treatment with the antioxidant, NAC.  For example, attenuation of the ROT mediated activation of p38 MAPK by NAC and resistance of the (SH)_2_Trx-ASK1 complex to dissociation in response to ROT treatment is consistent with our proposed mechanism that ROS from ETC dysfunction activates the p38 MAPK pathway via the dissociation of this complex.  We propose, therefore, that this mechanism is a component of the physiological interactions that define the overall mechanism of resistance to oxidative stress.

Our past studies have shown that aging tissues develop a state of chronic stress and that this occurs in the absence of extrinsic challenges [4,7,8].  This is indicated by the increased basal levels of (a) stress response genes, *e.g.,* the acute phase response genes; (b) the transcription factors that target these genes in aged tissues [4,7]; and (c) the demonstration that the basal levels of the p38 MAPK and SAPK/JNK signaling pathways are also up regulated with age [10,14].  Thus, we proposed that the mechanism of the age-associated increase in activity of stress response genes is a consequence of the stabilization of the elevated basal level of activity of the p38 MAPK and SAPK/JNK pathways and that the sustained and increased level of activity of these stress response pathways may be due to the alteration of the ASK1: (SH)_2_Trx-ASK1 ratio in response to the chronic increase in ROS production (Figure 1).  Our present studies with Ames dwarf fibroblasts derived from young and old mice suggest that ROS generated by ETC dysfunction may play a role in the mechanism of increased basal activity of p38 MAPK and SAPK/JNK stress signaling pathways in the aged tissues.  We attribute this to the chronic increase in ROS generated by mitochondrial dysfunction which regulates the ratio of ASK1 **: **(SH)_2_Trx-ASK1. Thus, the levels of Trx in the oxidized or reduced state are dependent on the effects of aging on the activity of thioredoxin reductase.  Based on our results we propose that (a) the ratio of ASK1 : (SH)_2_Trx-ASK1 shifts toward the dissociation of the complex, which elevates the pool of free ASK1 in aged tissue;   (b) this is the mechanism that causes an increased basal level of activity of the p38 MAPK and SAPK/JNK pathways in aged tissues, and (c) the aged tissues become less resistant to oxidative stress (Figuer 1).  These physiological processes would explain the progressive loss of resistance to ROT we observed in both wild-type and dwarf cells.

Our hypothesis is also supported by the observations that the Snell and Ames dwarf mutants are resistant to oxidative stress [35,37].  The combined increase in the level of (SH)_2_Trx-ASK1 complex and corresponding decrease of free ASK1 may account for the decreased activity of down stream targets and be indicative of their resistance to oxidative stress.  For example, the decreased activity of the components of the p38 MAPK pathway, MKK3 kinase activity, nuclear P-p38 kinase activity and ATF-2 activity in the Snell and Ames mice suggests a sustained lower level of activity associated with the response to oxidative stress in both young and aged dwarf mice [13].  Furthermore, the fact that the level of reduced thioredoxin is significantly higher in dwarf cells is consistent with the higher complex levels, lower stress signaling activity and resistance to oxidative stress.

Interestingly, since p38 MAPK is a master regulator of the activity of many genes, our proposed mechanism implies that the (SH)_2_Trx-ASK1 regulation of p38 MAPK activity should have multiple gene targets associated with resistance (and sensitivity) to oxidative stress.  This is consistent with the observation that ASK1 is selectively required for sustained activation of the p38 MAPK and SAPK/JNK pathways induced by oxidative stress [20].  This was demonstrated in ASK^(-/-) ^embryonic fibroblasts in which the H_2_O_2_ and TNF-mediated sustained activation of p38 MAPK and SAPK/JNK is lost.  These cells also exhibit elevated levels of the (SH)_2_Trx-ASK1 complex [20].  We propose, therefore, that the elevated level of the complex in Ames fibroblasts attenuates ASK1 activity thus mimicking the resistance to oxidative stress shown by the ASK1^(-/-)^ embryonic fibroblasts.  Furthermore, the elevated (SH)_2_Trx-ASK1 levels and attenuated p38 MAPK activity in livers of Snell dwarf mice [13] suggests that this may contribute to the resistance to oxidative stress exhibited by these mice.  At the same time, the constitutively elevated and sustained p38 MAPK activity in the wild-type aged livers may be due to the increased pool levels and activity of the uncomplexed ASK1.  Our hypothesis predicts that treatment of aged wild-type C57BL/6 mice with antioxidants may reverse the elevated endogenous levels of p38 MAPK and SAPK/JNK activity and increase resistance to oxidative stress.

Resistance to oxidative stress is an important physiological factor in the development of Snell and Ames dwarf longevity [35,37].  Our results suggest that the attenuation of the stress response signaling pathways (p38 MAPK and SAPK/JNK) in these mice may be a physiological indication of their resistance to oxidative stress.  Since the p38 MAPK can be activated by ROS generated by ETC dysfunction, the decreased state of oxidative stress in young and aged Snell and Ames mice correlates with their lower level of endogenous p38 MAPK activity.  Thus, the ratio of oxidized **: **reduced Trx which determines the activity of both ASK1 and the downstream components of the p38 MAPK pathway suggests a lower level of endogenous oxidative stress in the dwarf mouse.  This proposal is further supported by the higher levels of reduced thioredoxin.

Our studies raise the question of whether the regulation or elevation of (SH)_2_Trx-ASK1 levels due to the redox status of Trx may be a part of the mechanism of resistance to oxidative stress.  For example, activation of p38 MAPK in ASK-1^(-/-)^ embryonic fibroblasts by H_2_O_2_ and TNF is abolished in these cells which are resistance to H_2_O_2_-and TNF-induced apoptosis [20].  ASK-1 activity is, therefore, required for the sustained activation of p38 MAPK and SAPK/JNK by these ROS generating factors. Thus, the decreased ASK1 pool level of Snell dwarf mice (*in vivo*) and of the Ames dwarf fibroblasts (*in vitro)*, as well as the elevated levels of reduced thioredoxin and (SH)_2_Trx-ASK1 complex may contribute to the resistance to oxidative stress in these long-lived mice.  Our hypothesis is supported by the observation that Snell dwarf fibroblasts are resistant to oxidative stress generated by UV light, heavy metal (Cd), H_2_O_2_, paraquat and heat [37,42].

The physiological resistance to oxidative stress is a complex and likely integrated biological process.  The Ingenuity analysis of p38 MAPK protein-protein interactions shows an extensive number of genes whose activities are potentially important for the response and resistance to oxidative stress.  Both the up- and down-regulation of the p38 MAPK pathway activity by the ROS-mediated regulation of the level of (SH)_2_Trx-ASK1 complex may represent the mechanism of an integrated simultaneous regulation of multiple genes whose combined levels of activity define the physiological status that confers resistance to oxidative stress and longevity.

Trx can exert its protective functions either directly as an antioxidant by reducing the ability of oxidized Trx peroxidase to scavenge ROS, such as H_2_O_2 _, or indirectly by binding to signaling components and modulating their functions.  The complexing of Trx with the N-terminus of ASK-1 determines the ROS mediated regulation of activity of the p38 MAPK and SAPK/JNK stress response pathways [18].   Since the levels of reduced Trx and Trx bound to ASK-1 are much higher in both young and aged Ames fibroblasts and in Snell dwarf livers [13] than in their age-matched controls, we speculate that the dwarf tissues are in a more reduced state than their age-matched control livers.  Furthermore, the ability to maintain a lower level of stress activated p38 MAPK activity, which occurs throughout the life span of the dwarf, supports the hypothesis that its longevity may be associated with its redox state.  Our studies provide further support of the hypothesis that the age-associated increase in endogenous ROS generated by mitochondrial dysfunction may be a major factor in the sustained increase in stress response activity in aged tissue and in the development of age-associated physiological characteristics.

## Experimental procedures

Animals and tissues . The initial breeding pairs of wild-type and Ames dwarf mice were obtained from The Jackson Laboratory.  The mice were bred at the University of Texas Medical Branch and maintained in accordance with the institutional guidelines and were treated in accordance with UTMB-IACUC-approved protocols.  The genotypes of the mice have been determined and described [45].  Mouse tails were collected from wild-type dwarf males of 3 different age groups:  Young (3-6 months old), Middle aged (10-12 months old) and Old (21-24 months old).

Tail fibroblast isolation. The procedures described by Salmon et al. [42] were followed with some modifications.  Tails, excised from the mice after de-capitation, were kept in ice-cold PBS and then rinsed with 3 changes of 70% alcohol.  The distal half of the tail was cut into small pieces and minced with a single-edged razor blade in a pool of DMEM containing 15% fetal bovine serum plus 1X antibiotic and antimycotic solution (Gibco) in a 100 mm culture dish. The minced tail pieces were incubated with 2.5 ml of collagenase type II solution (400U/ml, Gibco) for 4-6 hours in a 37^o^ C incubator with 5% CO_2_ in air.  The cells were split six-fold 7-10 days after initiation of cultures to generate 1^st^ passage cells.  The 1^st^ passage cells were cultured for another 7-10 days; some of them which were split and seeded in 12-well culture dishes were used to generate growth curves.  The remaining cells were further expanded to 2^nd^ and 3^rd^ passages of cells for studies with ROT, 3-NPA, AA and H_2_O_2_.

Cell cultures and treatments. The tail fibroblasts were cultured in DMEM (Gibco or CellGro) containing 15% fetal bovine serum (Hyclone) and supplemented with antibiotic plus antimycotic solution (Gibco).  For ROT, 3-NPA, and AA (Sigma) treatment, the cells, at passage 3 or 4, were seeded in 100 mm^2^ culture dishes and cultured for 4-5 days until they reached approximately 70% confluence.  A day prior to inhibitor treatment, the medium was replaced with DMEM containing 0.5% FBS and antibiotics.  The cells were treated with 5 μM or 20 μM ROT, or with a series of ROT concentrations ranging from 20 μM to100 μM for 30 min; with 5 mM or 20 mM 3-NPA, or 50 μM AA for 30-, 60-, and 120-min.; with 0.2-1.0 mM, or 0.5-4.0 mM of H_2_O_2_ for 30 min.  The cells were pretreated with 20 mM of N-acetyl cysteine, (NAC; Sigma) for 30 min prior to the addition of ROT.  Protease inhibitors and phosphatase inhibitors were added as described [46].

Growth curves of fibroblasts. Fibroblasts (3 x 10^4^) from 3 different wild-type and Ames dwarf mice of young (3-6 mos), middle aged (10-12 mos) and old (21-24 mos) mice at passage 3 were plated in each well of a 12-well plate. The cells were harvested  at passage 3 or 4 and counted every day for 8 days. For the growth curves of fibroblasts in the continuous presence of rotenone, the same number of young wild-type and dwarf fibroblasts (40 x 10^4^/dish), at passage 4 were plated in a 100 mm cell culture dish and 20 μM of ROT was added to the treated groups of cells.  The cells were harvested and counted daily until the untreated wild-type cells become confluent in the dishes at the 4^th^ day.

Preparation of cellular extracts . Fibroblasts were washed 3x with ice-cold TBS (Tris-buffered saline), and scrapped from the dish by using a rubber policeman (Fisher).  The cells were centrifuged in a Beckman J6B at 400 g for 5 min at 4^o^C.  The extracts were prepared following the protocols described [45].  Protein concentrations were determined using the Bio-Rad protein assay reagent and bovine serum albumin as a standard (Bio-Rad).

Western blot analyses and immunoprecipitation assays. Fibroblast cytosolic protein (30 μg) was resolved on a precast 4-20% gradient SDS-PAGE (Lonza) or a 4-20% Criterion SDS-PAGE (Bio-Rad) and transferred onto an Immobilon PVDF membrane (Millipore; Bedford, MA). After primary and secondary antibody incubations, the proteins were detected according to the procedures recommended by the manufacturers using the Supersignal kits from Pierce (Rockford, IL) or Immobilon Western kit (Millipore).  In the cases where phosphorylation levels were measured, the membranes were probed with the antiphospho antibody first and the same membranes were washed and stripped, and probed with the corresponding antibodies to detect the levels of total proteins.  All the primary antibodies were purchased from Santa Cruz Biotechnology (Santa Cruz, CA) except for   P-p38 MAPK (#9211), p38 MAPK kinase assay kit (#9820; Cell Signaling Technology, Beverly, MA).  The bands detected by either antibody or antiphospho-antibody were imaged in a MultiImage Light Cabinet (Alpha Innotech) and the intensity of the image was measured.

Immunoprecipitation assays used to measure the pool levels of Trx bound to ASK1 were performed following the procedure provided by the manufacturer (Sigma, St. Louis, MO).  Briefly, 200 μg of pooled protein from 3 samples was precleared with proteon A conjugated agarose beads without antibody for 2 hr and then incubated with 4 μl of antibody at 4^o^C overnight. Protein A-conjugated agarose beads (30 μL) was added and incubated for additional 2-4 hr.  The beads were washed 4x with PBS and the eluted protein was precipitated by boiling for 5 min.  The precipitated protein was collected from the supernatant after boiling for 5 min.  The supernatant was resolved on a precast SDS-gel and transferred, and the membranes were processed as a regular Western blot.  The negative control tubes which did not contain proteins were processed in the same manner.

Determination of levels of reduced thioredoxin in fibroblasts. To detect the endogenous level of reduced thioredoxin in young wild-type and dwarf fibroblasts we added the thiol-interacting reagent, 4-acetamido-4'-maleimidyl stilbene-2,2'-disulfonic acid (AMS; Molecular Probes) to 100 μg of cell extracts at a final concentration of 15 mM in 20 mM Tris (pH 8.0) and incubated for 2 hrs at room temperature.  The reaction was terminated by adding SDS running buffer and the mixture was boiled for 5 min. Aliquots (15 μl) of samples were loaded onto a 15% SDS-PAGE, and the reduced form of thioredoxin was detected using thioredoxin antibody in a regular Western blot analysis.

*In Vitro *kinase assay. The *in vitro* p38 MAPK kinase assay was performed by modification of the procedures described by the manufacturer of the p38 MAPK assay kit (Cell Signaling Technology; Beverly, MA).  Fibroblast extract (200 μg total protein/reaction) was mixed with 20 μl of immobilized phosphor-p38 MAPK slurry in 1x lysis buffer, and incubated with constant rotation overnight at 4^o^C.  The immunoprecipitated beads were washed 2x with lysis buffer and 2x with kinase buffer, and then resuspended in 50 μl of kinase buffer containing 1 μg of ATF-2 fusion protein and 4 μM ATP.  The kinase reaction was carried out at 30^o^C for 25 min, and terminated by addition of 25 μl 3x SDS sample loading buffer.  The reaction mix was boiled for 5 min and the supernatant was collected and loaded on a precast 4-20% SDS-PAGE.  The gel was blotted onto a PVDF membrane and processed as described for Western blots using Supersignal West Pico detection kit.  The level of ATF-2 phosphorylated by p38 MAPK was determined using an antiphospho-ATF-2 antibody. The intensity of the phosphorylated ATF-2 band represents the relative p38 MAPK kinase activity precipitated by antiphospho-p38 MAPK antibody.

Statistical analysis . Statistical analyses were performed for age-matched comparisons with the single dependent variable being the Ames dwarf mutants.  The normalized values of protein and phosphoprotein bands or the average numbers of cells were analyzed using the two-tailed *t*-test to test the difference in means between age-matched groups at a significance level of 0.05.  The symbols (*) indicate statistical significance for the values represented by the bar or time point.
